# Impact of KRAS, BRAF and PI3KCA mutations in rectal carcinomas treated with neoadjuvant radiochemotherapy and surgery

**DOI:** 10.1186/1471-2407-13-200

**Published:** 2013-04-23

**Authors:** Olfa Derbel, Qing Wang, Françoise Desseigne, Michel Rivoire, Pierre Meeus, Patrice Peyrat, Mattia Stella, Isabelle Martel-Lafay, Anne-Isabelle Lemaistre, Christelle de La Fouchardière

**Affiliations:** 1 Department of Medical Oncology, Centre Léon Bérard, 28 rue Laennec, Lyon, 69008, France; 2 Department of Biopathology, Centre Léon Bérard, 28 rue Laennec, Lyon, 69008, France; 3 Department of Surgery, Centre Léon Bérard, 28 rue Laennec, Lyon, 69008, France; 4 Department of Radiotherapy, Centre Léon Bérard, 28 rue Laennec, Lyon, 69008, France; 5 Department of Anatomopathology, Centre Léon Bérard, 28 rue Laennec, Lyon, 69008, France

## Abstract

**Background:**

Conventional treatment for locally advanced rectal cancer usually combines neoadjuvant radiochemotherapy and surgery. Until recently, there have been limited predictive factors (clinical or biological) for rectal tumor response to conventional treatment. *KRAS*, *BRAF* and *PIK3CA* mutations are commonly found in colon cancers. In this study, we aimed to determine the mutation frequencies of *KRAS*, *BRAF* and *PIK3CA* and to establish whether such mutations may be used as prognostic and/or predictive factors in rectal cancer patients.

**Methods:**

We retrospectively reviewed the clinical and biological data of 98 consecutive operated patients between May 2006 and September 2009. We focused in patients who received surgery in our center after radiochemotherapy and in which tumor samples were available.

**Results:**

In the 98 patients with a rectal cancer, the median follow-up time was 28.3 months (4–74). Eight out of ninety-eight patients experienced a local recurrence (8%) and 17/98 developed distant metastasis (17%). *KRAS, BRAF* and *PIK3CA* were identified respectively in 23 (23.5%), 2 (2%) and 4 (4%) patients. As described in previous studies, mutations in *KRAS* and *BRAF* were mutually exclusive. No patient with local recurrence exhibited *KRAS* or *PIK3CA* mutation and one harbored *BRAF* mutation (12.5%). Of the seventeen patients with distant metastasis (17%), 5 were presenting *KRAS* mutation (29%), one *BRAF* (5%) and one *PIK3CA* mutation (5%). No relationship was seen between *PIK3CA*, *KRAS* or *BRAF* mutation and local or distant recurrences.

**Conclusion:**

The frequencies of *KRAS*, *BRAF* and *PIK3CA* mutations in our study were lower than the average frequencies reported in colorectal cancers and no significant correlation was found between local/distant recurrences and *KRAS*, *BRAF* or *PIK3CA* mutations. Future studies with greater number of patients, longer follow-up time and greater power to predict associations are necessary to fully understand this relationship.

## Background

Over the last decade, the management of colorectal cancer (CRC) has progressed faster than in any other gastrointestinal tumors [1]. These advances have been made especially in metastatic disease, with the introduction of targeted therapies in addition to chemotherapy and the development of metastasis surgery [2-5]. Improvements have also been made in the adjuvant setting with the introduction of the oxaliplatin-based chemotherapy regimen in stage III colon cancer [6]. Less progress has been made in the management of rectal cancer. Radiochemotherapy based on 5FU regimen, followed by total mesorectum excision (TME) represents the optimal combined treatment for locally advanced rectal cancer (defined as T3 and/or N+ disease) [7,8]. Neoadjuvant radiochemotherapy has been shown to reduce local recurrences and to increase pathological complete response compared with radiotherapy and surgery [9-11]. This preoperative modality is currently preferred to the postoperative one because of a significantly lower local recurrence rate, improved sphincter preservation and less toxicity [12,13]. Attempts to increase the benefit of radiochemotherapy have been tried, especially with the introduction of oxaliplatin in addition to capecitabine but finally, the 5FU based radiochemotherapy has remained the standard treatment for patients with locally-advanced rectal cancer [9,13-15]. The decision to use neoadjuvant radiochemotherapy is based on a pre-treatment tumor staging defining the T and the N stage with pelvic MRI and endorectal ultrasound. The tumor response is evaluated by the pathological examination of the operative specimen. It is well known that downstaging after radiochemotherapy has been shown to predict fewer recurrences and better prognosis [13,16]. However, the decision to use neoadjuvent radiochemotherapy is complex. First, the tumor response can be evaluated only after the pathological examination. Secondly, despite low local recurrence rates, patients with initially localized rectal cancer continue to have high mortality because of secondary metastases (15-35%). On the other hand, some patients may be over treated with radiochemotherapy. Therefore, many authors have tried to identify predictive factors to anticipate radiochemotherapy response. Currently, the best available methods to investigate improved outcomes in rectal cancer include accurate early assessment of tumor response with MRI and identifying predictive molecular tumor abnormalities.

*KRAS*, *BRAF* and *PIK3CA* mutations are commonly found in colorectal cancers. *KRAS* and *BRAF* genes can harbor oncogenic mutations that yield a constitutively active protein and are found in approximately 30–50% and 10–15% of CRC tumors, respectively [17,18]. Several studies have indicated that the presence of mutant *KRAS* in CRC tumors correlates with poor response to EGFR in a metastatic setting [5,18-20]. Furthermore, *BRAF* mutations have been incriminated as poor prognosis factors in metastatic CRC [21]. However, the impact of *KRAS* and *BRAF* mutations on clinical outcome of patients with locally advanced CRC are unknown. Regarding *PIK3CA*, a large cohort study has recently shown that *PIK3CA* mutation was associated with poor prognosis among patients with resectable stage I to III colon cancer [22]. Another large population-based study in colon cancer suggested that the activation of the PI3K/AKT or the RAS-RAF-MAPK pathway by mutation of at least one of the three genes predicted poor patient outcome, but the effect of mutations in *PIK3CA* alone was not discussed [17]. Another previous study of a small cohort of colorectal cancer patients reported that *PIK3CA* mutation is predictive of poor survival [23]. Recently, He et al. showed that *PIK3CA* mutations were strongly associated with a high risk of local recurrences in non irradiated stage I to III rectal cancer patients [24]. As their population was heterogeneous in tumor stage (I to III) and was not treated with combined modality therapy, we aimed to corroborate the mutation frequencies of *KRAS*, *BRAF* and *PIK3CA* in rectal cancer and to establish whether such mutations may be used as prognostic and/or predictive factors in multimodal treated rectal cancer patients. This study is the first to look at all three mutations in locally advanced rectal (not colorectal) cancer in patients treated with neoadjuvent chemotherapy and surgery.

## Methods

### Patients and tumor samples

The clinical records of all consecutively patients with locally advanced rectal carcinoma (clinical T3 or T4 or node-positive) referred to the Centre Leon Berard between May 2006 and September 2009 were reviewed. The study was approved by the ethic committee of Leon Berard Center. Written informed consent was obtained for all patients.

The inclusion criteria were a confirmed diagnosis of rectal adenocarcinoma and available tumor sample. All patients gave their informed consent for this research. Diagnosis was established on the basis of histological features and was confirmed by immunochemical staining. Pathology procedures were standardized and quality controlled. Prior to treatment, a history and physical exam were completed for all patients as well as assessment of performance status, complete blood counts (CBCs), liver function creatinine, and serum carcinoembryogenic antigen. All patients underwent before treatment a rigid rectoscopy and a total colonoscopy. Tumor and nodal stage was evaluated with pelvic MRI and/or an endorectal ultrasound. Metastatic extension was eliminated with a chest- abdomen-pelvis computed tomography. Clinical tumor staging was finally defined with the “i” (MRI) or “u” (ultrasound) tumor-node-metastasis (TNM) classification. Clinical examination and CBCs were repeated every week during radiochemotherapy. Four weeks after the end of radiochemotherapy, clinical tumor stage was re-evaluated with pelvic MRI and CT. After surgery, patients were assessed every 3 months during the first two years and every 6 months during years 3 to 5.

Patients were treated with neoadjuvant radiochemotherapy and TME-surgery. Radiotherapy consisted on 45 to 50 Gy in 25 fractions of 1.8 to 2Gy with concurrent intravenous 5FU or capecitabine. Oral capecitabine 800 mg/m2 twice daily was started on the first day of radiotherapy and given 5 days per week during radiotherapy. When used, infusional 5FU was given at a dose of 350 mg/m2/d from Monday to Friday with leucovorin at a dose of 20 mg/m2/d. Surgery was planned 6 weeks after the end of preoperative radiochemotherapy. Total mesorectal excision was performed according to a standardized technique.

### DNA extraction and mutation analysis

DNA was amplified with specific primers for exons where "hot-spot" mutations are located. DNA was extracted from FFPE primary tumor samples using QIAamp DNA FFPE Tissue Kit (Qiagen, Hilden, Germany). Mutation status of *KRAS* gene (exon 2 and 3), *PIK3CA* gene (exon 9 and exon 20), *BRAF* gene (exon 15), was investigated by PCR amplification followed by direct sequencing using ABI 3730 automated sequencer (life Technologies). The oligo sequences of primers used for analyses are available upon request.

### Statistical analysis

All statistical analyses were done with SPSS statistical software (version 15.0 for Windows, SPSS, Inc). χ2 test and Fisher's exact test were used to compare proportions. Recurrences and survival analyses were done using the Kaplan-Meier method with time of surgery as entry date. Logrank testing was used for comparison of groups.

## Results

### Patient characteristics

Ninety-eight consecutive patients treated at the Centre Leon Berard, Lyon, France for an advanced rectal cancer between May 2006 and September 2009 met the inclusion criteria. Locally advanced rectal cancer was defined as T3 and/or N+ disease with pelvic MRI and/or endorectal ultrasound. Median follow-up was 28.3 months (4–74). Patient’s characteristics are listed in Table 1.

**Table 1:** Clinical and molecular characteristics of 98 patients with rectal carcinoma

**Clinical and molecular characteristics**	**No patients *n*=98 (%)**
Sex	
Male	50 (51)
Female	48 (49)
Age. y median	68 (35–88)
Distance to anal verge. cm	
≥10	25 (25.5)
5≤ <10	26 (26.5)
<5	40 (41.8)
Not avaibale	7 (7.2)
Type of resection	
Low anterior	8 (8.1)
Abdominoperineal	90 (91.9)
CRM	
Negative	88 (89.8)
Positive	3 (3.1)
Not avaiballe	7 (7.1)
Differentiation	
Well	15 (15.1)
Moderate	51 (52.1)
Poor	10 (10.3)
Not avaibale	22 (22.5)
TNM stage	
I	6 (6.1)
II	86 (87.8)
II	6 (6.1)
KRAS mutation	
Yes	23(23.5%)
No	75 (76.5)
BRAF mutation	
Yes	2 (2%)
No	96 (98%)
PIK3CA mutation	
Yes	4 (4%)
No	94 (96%)
Local recurrence	
Negative	90 (91.8)
Positive	8 (8.2)
Distant metastasis	
Negative	81 (82.6)
Positive	17 (17.4)

### Pathologic characteristics

We correlated the *KRAS, BRAF* and *PIK3CA* genotypes with clinicopathological features of CRC, including primary tumour location, histological findings, and sites of metastases. There was no correlation between mutations and clinicopathologic features, including age, gender, tumor location, type of resection, circumferential margin (CRM), differentiation grade, lymph node and TNM stage.We also investigated whether *KRAS*, *BRAF* or *PIK3CA* mutations may confer radioresistance and reduced response to CRT. There was no difference in residual tumor status or in response to chemoradiation as assessed by tumor downstaging, based on mutations status.

### Mutation analysis

**Figure 1:**
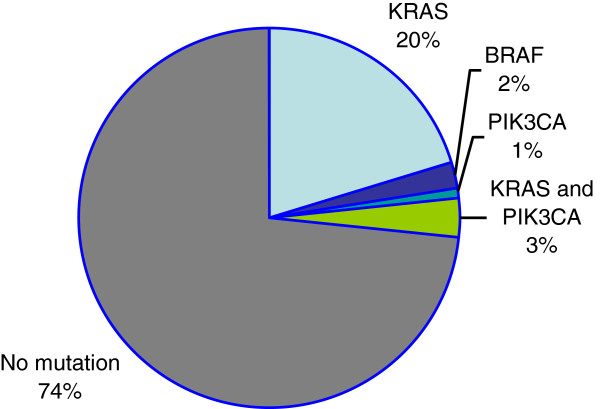
The distribution of mutations is illustrated in a pie chart.

*KRAS*, *BRAF* and *PIK3CA* were identified respectively in 23 (23.5%), 2 (2%) and 4 (4%) patients. The most frequent mutation at *KRAS* was G13D which accounted for 43% of *KRAS* mutations (10/23). The codon 12 mutations were the G12D (5/23), G12V (4/23), G12S (2/23), G12R (1/23) and G12C (1/23). *BRAF* V600E mutation was identified in one patient (50%). Mutations are summarized in Table 1 and the distribution of the mutations is shown in Figure 1. As described in previous studies, mutations in *KRAS* and *BRAF* were mutually exclusive.

### Correlation with local and metastatic recurrences

With a median follow-up time of 28.3 months (4–74), 8 patients experienced a local recurrence (8%) and 17 developed distant metastasis (17%). No patient with local recurrence exhibited *KRAS* or *PIK3CA* mutation and one harbored *BRAF* mutation (12.5%). Among the seventeen patients (17%) with distant metastasis, 5 were harboring *KRAS* mutation (29%), one *BRAF* (5%) and one *KRAS* mutation (5%). Survival analyses compared results for patients with each mutation, patients with at least one of the 3 mutations versus those who had no mutations (Tables 2 and 3).

**Table 2:** Mutational status and local recurrence

	**Local recurrence**				**Patients**	
	**No**		**Yes**			
	**N=90**		**N=8**		**N=98**	
***KRAS *Mutation**						
No	67	(74.4%)	8	(100.0%)	75	(76.5%)
yes	23	(25.6%)	0	(0.0%)	23	(23.5%)
***BRAF *Mutation**						
No	89	(98.9%)	7	(87.5%)	96	(98.0%)
yes	1	(1.1%)	1	(12.5%)	2	(2.0%)
***PIK3CA *Mutation**						
No	86	(95.6%)	8	(100.0%)	94	(95.9%)
yes	4	(4.4%)	0	(0.0%)	4	(4.1%)
**All mutations**						
At least one mutation						
No	65	(72.2%)	7	(87.5%)	72	(73.5%)
yes	25	(27.8%)	1	(12.5%)	26	(26.5%)
Nb of mutations						
0	65	(72.2%)	7	(87.5%)	72	(73.5%)
1	22	(24.4%)	1	(12.5%)	23	(23.5%)
2	3	(3.3%)	0	(0.0%)	3	(3.1%)

**Table 3:** Mutational status and metastatic recurrence

	**Metastatic recurrence**				**Patients**		**Test**
	**No**		**Yes**				
	**N=81**		**N=17**		**N=98**		
***KRAS *mutation**							
Non	63	(77.8%)	12	(70.6%)	75	(76.5%)	
Yes	18	(22.2%)	5	(29.4%)	23	(23.5%)	
***BRAF *mutation**							Fisher Exact P = 0.318
Non	80	(98.8%)	16	(94.1%)	96	(98.0%)	
Yes	1	(1.2%)	1	(5.9%)	2	(2.0%)	
***PIK3CA *mutation**							Fisher Exact P = 0.539
Non	78	(96.3%)	16	(94.1%)	94	(95.9%)	
Yes	3	(3.7%)	1	(5.9%)	4	(4.1%)	
**All mutations**							
At least one mutation							Fisher Exact P = 0.377
No	61	(75.3%)	11	(64.7%)	72	(73.5%)	
Yes	20	(24.7%)	6	(35.3%)	26	(26.5%)	
Nb of mutations							Fisher Exact P = 0.462
0	61	(75.3%)	11	(64.7%)	72	(73.5%)	
1	18	(22.2%)	5	(29.4%)	23	(23.5%)	
2	2	(2.5%)	1	(5.9%)	3	(3.1%)	

No relationship was seen between PIK3CA, *KRAS* or *BRAF* mutation and local or distant recurrences and no longer with overall survival (all p< 0.01).

## Discussion

Despite the recent advances in the management of metastatic colorectal cancer (mCRC) over the last few years, this disease remains a major public health problem in the world [1]. Its prognosis has been improved, but recurrences remain a clinical challenge. Local recurrences are a critical issue in rectal cancer and several tumor characteristics are associated with local recurrence, including depth of tumour invasion into and beyond the bowel wall, number of lymph nodes involved by tumor cells, extramural venous invasion, involvement of the circumferential resection margin (CRM) and the tumor location within 10 cm from anal verge. These factors may be used in therapeutic decisions regarding administration of (neo) adjuvant treatment [25]. However, patients with similar clinicopathologic characteristics still display a large variation in prognosis, suggesting that the biology of the tumor may be responsible for the difference. The detection of biological predictive markers may improve the selection of patients who will benefit from (neo) adjuvant treatment and spare patients who will only experience side effects. The MAPK pathway plays a major role in cell proliferation and is involved in up to 30% of CRC [26]. Both *KRAS* and *BRAF* are the members of this signalling pathway and are known to be activated by oncogenic mutations. *KRAS* mutations, are reported in 19 to 48% of patients with rectal cancer [1,24-27], whereas *BRAF* mutations are found in 0 to 12% of RC patients [28,29]. The mutation frequency reported in our study (23% for *KRAS* and 2% for *BRAF*) are in concordance with those reported in literature. The rarity of V600E *BRAF* mutations in rectal cancer has been previously described in reports from Di Nicolantonio et al. [30] who found 1 V600E allele in 43 rectal samples and Fransen et al. [28] who even found 2 mutations in 55 rectal cancers.

In our study, we identified a 4% incidence for *PIK3CA* mutations, which is lower than the 10% to 30% reported in colon cancer. The first study in colorectal cancer reported that *PIK3CA* mutations were significantly associated with poor survival [23]. They further identified *PIK3CA* mutations as the only independent and significant prognostic factor for relapse-free survival in stage II to III patients. However, there were only 18 *PIK3CA*-mutated tumors and the number of deaths was not reported. Another study has shown that the presence of at least one mutation in *PIK3CA*, *BRAF*, or *KRAS* genes predicts poor survival in a population-based colon cancer samples, however, the effect of *PIK3CA* mutations on survival, independently of clinical and other molecular predictors of outcome, was not described [31]. In a recent study, Ogino et al. examined the prognostic significance of *PIK3CA* mutations among 450 patients who had undergone a curative resection of colon cancer. They found that *PIK3CA* mutations were associated with a decreased survival. The “poor prognosis” effect of *PIK3CA* mutations seemed to be robust and consistent across most strata of clinical and tumoral predictors of patient outcome, but this effect seems to be limited to patients with *KRAS* wild-type tumors [22]. Additionally, these mutation studies make no distinction between rectal and colic cancer. A recent study determined the prognostic value of *PIK3CA* mutations in 240 non- irradiated resectable stages I to III rectal cancer patients from the Dutch TME trial. *PIK3CA* mutations were identified in 19 (7.9%) of the 240 patients and revealed a strong association with an increased local recurrence rate. Moreover, tumors with *PIK3CA* mutations showed a tendency to develop local recurrences more rapidly after surgery [24].

A second analysis by He et al. was published in 2010 concerning the results in irradiated patients from the total mesorectal excision (TME) trial. In this population, they investigated whether *PIK3CA* mutant patients benefit from preoperative radiotherapy. Although the difference was not statistically significant, it suggests that *PIK3CA* mutant patients seem to benefit from irradiation in preventing LR [32].

Several studies have focused on *KRAS* mutation and radioresistance, with controversial conclusions. Michelassi et al. found that tumor downstaging was associated with *KRAS* wild type tumors [33] and Grana et al. reported that *KRAS* mutations potentially mediate resistance to ionizing radiation [34]. Other studies reported no change in downstaging by *KRAS* status [29,35,36] and even when adjusting the groups according to the codon which carried the mutation, failed to predict response to preoperative CT/RT. In a recent study, Davies showed that *KRAS* mutational status was not associated with radiosensitivity using more modern sequencing technology in a larger number of patients than previously described [37]. Interestingly, they reported that activation of AKT and ERK is correlated with response to radiation therapy [37].

## Conclusions

In summary, our retrospective study failed to confirm a significant correlation between *KRAS*, *BRAF* or *PIK3CA* mutations as predictive factors of local or distant recurrence, following preoperative RT/CT. The exact effects of *KRAS*, *BRAF* and *PIK3CA* mutations on recurrence require further study with analyses of a larger patient population because the number of relapse events was very small and may represent a sample bias. Finally, the follow-up period was probably too short to draw definitive conclusions.

## Competing interest

The authors declare that they have no competing interests.

## Authors’ contributions

All authors collected data, reviewed the draft, provided comments or substantive revisions, and approved the final manuscript. All authors read and approved the final manuscript.

## Pre-publication history

The pre-publication history for this paper can be accessed here:

http://www.biomedcentral.com/1471-2407/13/200/prepub
